# Use of High-Fidelity Simulation as an Adjunct to Basic Life Support Training To Promote Team-Based Resuscitation Skills: A Mixed-Methods Pilot Study

**DOI:** 10.7759/cureus.62719

**Published:** 2024-06-19

**Authors:** Daniel J Berger, Lauren Lum, Ryan Shercliffe, Elizabeth Sinz

**Affiliations:** 1 Emergency & Internal Medicine, Virginia Commonwealth University Health System, Richmond, USA; 2 Resuscitation Sciences Training Center, Penn State Health Milton S. Hershey Medical Center, Hershey, USA; 3 Anesthesiology and Critical Care, West Virginia University, Morgantown, USA

**Keywords:** bls, cardiac arrest, cpr, resuscitation training, team-dynamics, high-fidelity simulation, basic life support

## Abstract

Introduction

The 2020 American Heart Association’s (AHA) Basic Life Support (BLS) curriculum focuses on cardiac arrest resuscitation with one or two rescuers, providing only limited opportunities to develop higher-level skills such as leadership, communication, and debriefing. This mixed-methods pilot study evaluated whether supplementing the traditional Heartcode BLS course with a high-fidelity teamwork simulation session improved mastery of these higher-level skills.

Methods

Twenty-four first-year medical students completed the pilot training during sessions offered in February and May of 2023. The program included the traditional AHA Heartcode BLS course, which ranges from two to four hours, and includes both online and in-person skills components. This was followed by a 90-minute high-fidelity simulation session consisting of two simulated resuscitations separated by a student-led plus/delta debriefing. Facilitators then debriefed the entire activity. Students completed an anonymous online survey that used a 0-10 slider scale to attribute their perceived proficiency for specific skills to the initial BLS course or the teamwork simulations and provided qualitative feedback.

Results

Twenty-one students (87.5%) completed the follow-up survey. Students credited their proficiency in technical skills (e.g., “Chest Compressions”) to both sessions equally, but proficiency in higher-level skills, such as leadership, communication, and teamwork, was predominantly credited to the simulation. Additionally, students reported that the teamwork simulation promoted realism and increased self-efficacy.

Conclusion

Team-based resuscitation simulations using high-fidelity equipment augmented the AHA BLS course by promoting perceived competence in team dynamics domains and increasing students’ self-efficacy for participating in real hospital-based resuscitations. Studies with larger sample sizes and objective data should be performed, and the use of similar resuscitation simulations or the development of a formal team-based BLS certification course should be considered.

## Introduction

Basic life support (BLS) is the standard treatment for patients experiencing cardiac arrest, with a high chest compression fraction and early defibrillation shown to improve outcomes [1]. The American Heart Association (AHA) BLS course trains participants to deliver chest compressions, ventilations, and defibrillation using an AED, primarily focusing on one- and two-rescuer CPR with an optional team dynamics activity [2, 3]. However, additional challenges faced by cardiac arrest responders include the logistics of hospital room configurations and interdisciplinary team interactions. Many of these aspects are highlighted in the AHA’s Advanced Cardiac Life Support (ACLS) course, which focuses on resuscitation team dynamics, including communication and leadership. This discrepancy in training creates a potential knowledge gap for BLS-trained responders who may participate in team-based resuscitations.

At the Pennsylvania State College of Medicine (PSUCOM), medical students are required to obtain BLS, but not ACLS, certification prior to their clinical duties. While this trains them in the technical skills of CPR, it does not provide significant experience in resuscitating a patient in a realistic inpatient setting or as part of a larger resuscitation team. To address this knowledge gap, our team piloted a high-performance team dynamics simulation session designed to supplement the traditional Heartcode BLS course for first-year medical students.

## Materials and methods

Description of training activity and participants

**Figure 1:**
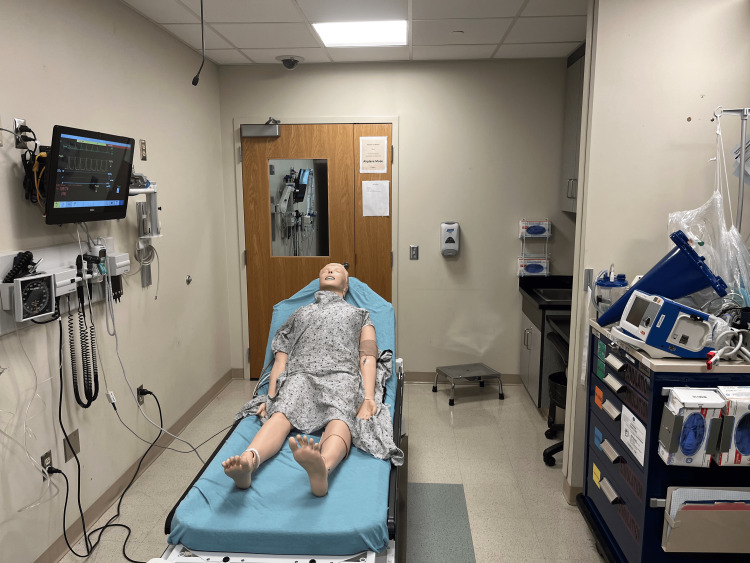
Initial Room Setup for High-Fidelity Team Simulation Activity

We recruited first-year medical students enrolled at Penn State Health's Resuscitation Sciences Training Center (RSTC) by email to participate in the training session. They first completed the AHA’s blended Heartcode BLS course. The Heartcode BLS course consists of a one-to-two-hour online learning component and an additional one-to-two-hour in-person skills session (American Heart Association, Dallas, Texas). At RSTC, learners complete the hands-on skills portion in approximately one hour, and we include the optional team dynamics activity in our Heartcode sessions. However, due to the length of the course, this entails only one short session of team-based CPR and does not include the use of any high-fidelity equipment. Following completion of the Heartcode course, students were oriented to the hospital’s monitor-defibrillator (R Series Monitor Defibrillator, Zoll Medical Corporation, Chelmsford, Massachusetts), high-fidelity manikin (SimMan 3G, Laerdal, Wappinger Falls, New York), and monitoring equipment prior to beginning the first simulation session. A photograph of the initial room setup prior to the start of the high-fidelity session is shown in Figure 1.

**Figure 2:**
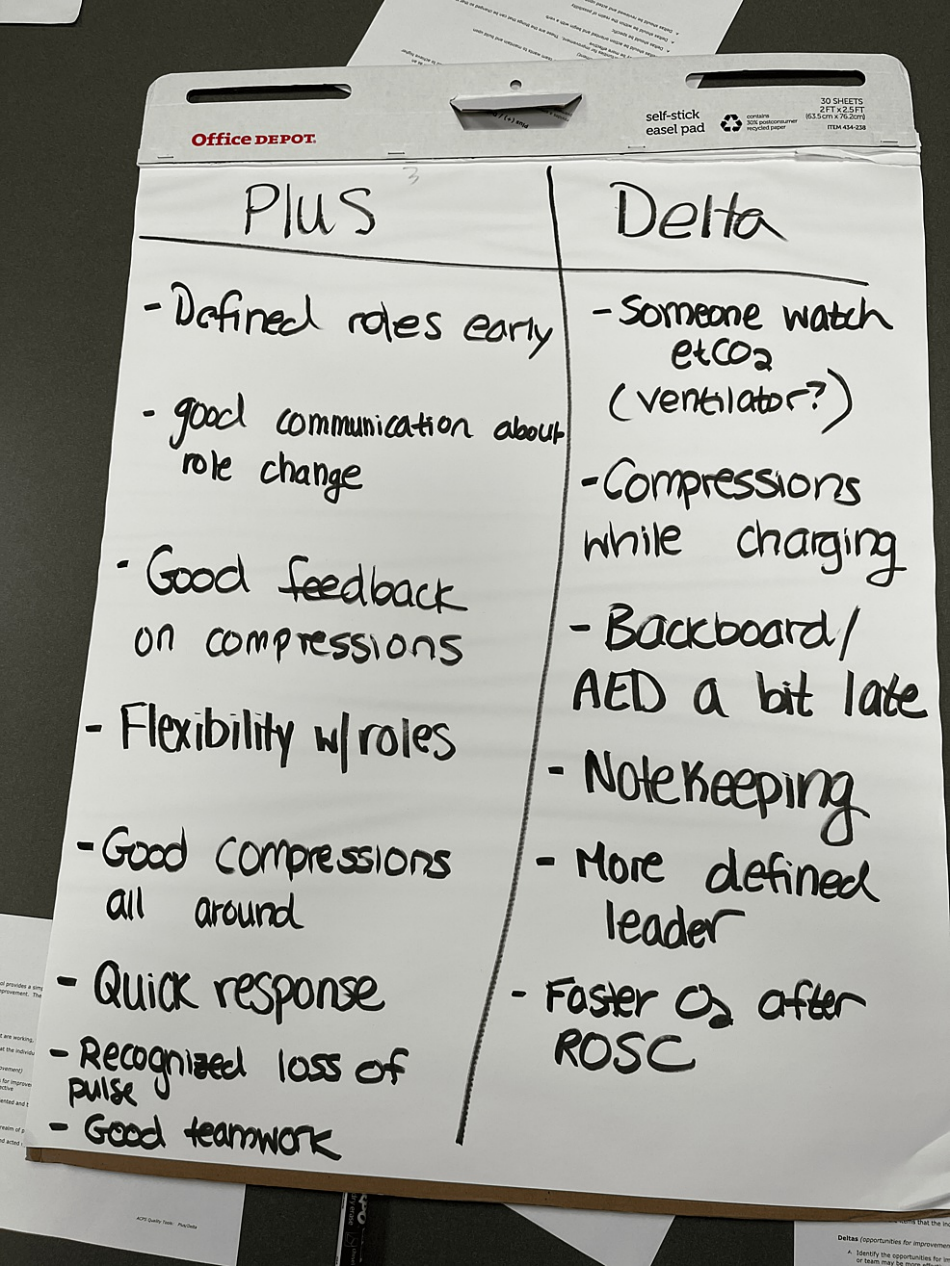
Sample Plus/Delta Created by Resuscitation Team During Self-Debrief

Students were provided with a brief clinical scenario to begin each simulation case and were directed to assess their patient and take any appropriate interventions. For the first case, teams of four to five students encountered a bradycardic patient who quickly developed ventricular fibrillation. Following the case, the team used standard plus/delta instructions (Plus/Delta, APCS Quality Tools) to self-debrief and formulate a performance improvement plan. A plus/delta created by one of the teams is shown in Figure 2. The team then completed a second case involving a patient who experienced respiratory arrest and subsequent cardiac arrest after being administered an opioid. Facilitators then debriefed students about the entire activity.

Study data and analysis

Students completed an anonymous online survey (Appendix) approximately one week after their session using REDCap [4]. Using slider scales, they identified at what point during the two sessions (Heartcode BLS or team-based simulation session) they achieved proficiency for various skills. The scales were automatically coded as numerical values ranging from 0 to 10, with zero indicating the participant attributed their proficiency entirely to the BLS course, 10 indicating attribution entirely to the team-based simulation, and five indicating equal attribution to both sessions. End-tidal CO2 (ETCO2) was included to validate the survey as it was only briefly mentioned during the Heartcode BLS course and emphasized during the simulation session. In addition to the quantitative responses, participants provided demographic information and answered open-ended questions. Students were incentivized to complete the surveys by the opportunity to enter into a drawing for a five-dollar gift card. Themes were extracted from the open-ended survey questions and excerpts from transcripts of the facilitated debriefs. Descriptive statistics and t-tests were generated using Microsoft Excel (Microsoft Corporation, Redmond, Washington). The project received a Not Human Subjects Research Determination from the Pennsylvania State University Institutional Review Board.

## Results

Twenty-four first-year medical students completed the activity. Their average age was 26 years (range: 22-37 years), 66% identified as women, and 66% had previously taken a BLS course. Twenty-one (87.5%) students completed the post-session follow-up survey.

Skills grouped together for analysis are shown in Table 1. Students indicated that their proficiency in rudimentary skills, such as “identifying a patient in need of CPR” and “performing chest compressions,” resulted almost equally from both sessions (average score 5.2, SD = 2.3). However, students credited the team-based simulation somewhat more (p < 0.002) than the BLS course for their proficiency in “real-time improvement skills” such as “use of a CPR coach to augment feedback” (average score 6.6, SD = 2.3). They credited the team-based simulation substantially more (p < 0.0001) for their proficiency in higher-level skills such as “actively participating as a member of a team” and “leading a team” (average score 8.3, SD = 1.3).

**Table 1:** Participant Attribution of Skills to BLS and Simulation Activity *Means are derived from 0-10 rating scales based on how participants attributed their skill proficiency, where 0 = skill proficiency acquired entirely from the AHA Heartcode BLS session, 10 = skill proficiency acquired entirely from the simulation session, and 5 = skill proficiency acquired equally from both sessions. CPR: cardiopulmonary resuscitation, AED: automated external defibrillator, ETCO2: end-tidal carbon dioxide, ROSC: return of spontaneous circulation, BLS: Basic Life Support, AHA: American Heart Association.

Skill Type	Mean*
Rudimentary skills:	5.2
Identify a patient in need of CPR (lacking responsiveness, breathing, and pulse).	5.1
Provide effective chest compressions.	4.9
Adequately ventilate a patient with a bag valve mask.	5.0
Correctly operate an AED.	5.8
Higher level skills:	8.3
Actively participate as a team member providing basic cardiopulmonary resuscitation.	8.6
Lead a team providing basic cardiopulmonary resuscitation.	8.2
Communicate with team-members using closed-loop communication.	8.3
Conduct a post-event debrief.	8.8
Create a plan to improve team performance for a future resuscitation.	8.6
Understand the defined team roles of a high-performance CPR team.	7.0
Real-time improvement skills:	6.6
Incorporate feedback data to improve CPR performance.	6.8
Use a CPR coach to augment feedback.	5.2
Provide corrective feedback to my team members.	7.4
Survey validation:	
Use ETCO2 to recognize ROSC.	8.9

During the facilitated debriefing, students reported that the use of high-fidelity equipment promoted realism and that the realistic setting increased their feelings of self-efficacy. They recognized the benefits of a post-event debriefing and subsequent performance improvement plan, tools they stated would be beneficial during their pre-clinical studies and future clinical rotations. Students also felt that the simulation training helped prepare them for situations with real patients by increasing their understanding of team roles, responsibilities, and communication, with 70% indicating that the simulation session should be required as part of their curriculum. Selected student comments are shown in Table 2.

**Table 2:** Selected Qualitative Comments From Learner Debrief and Post-session Survey

Selected learner comments
Benefits of realism: “The simulation gave me a better feel for what a code actually looks like as opposed to the normal BLS [course] that doesn't ‘feel’ real.” “Before it’s all theoretical, even those practice dummies, it’s like ‘ok this isn’t a person, I have no attachment to this whatsoever.’ But the scenario brings you closer to the point of it being more real, and when it’s more real you’ll ingrain it more in your head.” “It was more realistic, and because of that, it was more memorable.” “[After completing the scenario] maybe it’s a little less traumatic when it happens in real life.” “BLS class was definitely good for chest compression and airway, but that’s the foundation for putting it into a bigger picture which is in-hospital.” “The simulation provided a more realistic scenario to apply skills to, and an opportunity to reflect, improve our practice, and apply those skills readily.” “I felt like I was able to better get a grasp on how well my compressions are and how to work more efficiently in a room setting. There were small things like where to be and what wires would be in the way that wasn't as clear in just the [BLS] session.”
Confidence and self-efficacy: “Though the BLS class was helpful to gain the basic skills, being in a simulated environment allowed me to gain a little more comfort and familiarity with the different concepts. It made the idea of being in a real-life situation less frightening.” “[The simulation] increased my confidence to actually perform CPR, and it solidified it in my brain because I was actually able to run through the scenario.”
Roles & team dynamics: “I think for me, this was my first real time feeling what it was like to work as a team in the hospital.” “I think the team aspect was important. I know in the BLS class they said you needed to have closed loop communication, but [the simulation allowed] actually doing it in a more realistic setting.” “Knowing what each role does—like if someone told me I was a team lead, I might not know all of the responsibilities of a team lead, but now I have a better sense of that.” “Team dynamics, which are crucial to quality healthcare, could not have been taught by the BLS class alone.”
Debriefing: “I feel like we communicated more [...]. I don’t think we would have done that if we went straight into scenario two without talking, I don’t think we would have done most of those things.” “I feel like being left alone in the room [without] someone further along in their experience and training than us [...] forced us to ‘ok we’re it, so what are we going to do with this situation?’ And obviously it’s low stakes so it’s fine. But I feel like it helped [us] think about really clear communication.” “I felt like we can release some of that hesitancy by sitting here and talking about it and saying well that’s what we think went well and that’s what we think didn’t go well to try to be like ok maybe I’ll grab-err—are you going to grab that?”

## Discussion

This mixed-methods study evaluated whether a pilot simulation training session added to the standard AHA Heartcode BLS session would better prepare first-year medical students to actively participate in resuscitations. Students indicated that the high-fidelity simulation helped them become proficient in both rudimentary skills and higher-level team-based resuscitation skills, including leadership, beyond what they would have gained from the AHA BLS course alone. The repetitive learning exercises used in this pilot have been shown to be valuable for long-term memory formation and future skills performance [5].

Previous research has shown that recognition pathways produced and reinforced through simulation are stronger and more reliable than those developed through didactic learning alone [6]. The creation and strengthening of neural pathways is the basis of recognition-primed decision-making (RPDM), which promotes understanding of the intuition or “gut instinct” some healthcare providers exhibit when recognizing impending patient deterioration before it occurs [7]. When students are actively engaged in learning, such as through simulation, they more quickly recognize previously experienced errors and can identify possible solutions [6, 8]. Simulation provides a safe and controlled learning environment to reinforce RPDM pathways without any threat to patients. Simulation-based training also promotes a holistic understanding of the resuscitation process and a shared mental model by enabling learners to perform roles, such as leading a team, that they might not be eligible for in the clinical setting [9]. This may explain why medical students who participate in simulations have more real clinical experiences on the wards [10].

Participants attributed the development of their proficiency in higher-level skills, such as leadership, more to the team-based simulation than to the BLS course alone. This is an important finding, as previous studies of cardiac arrest resuscitation teams, both during simulation and in situ, reported associations between effective leadership and improved resuscitation performance [11-13]. Effective leadership has specifically been associated with complex skills such as higher compression fraction, as well as shorter pre-shock pauses and time to defibrillation [11].

A study conducted by Hunkier et al. found that medical student teams that received leadership instruction increased their leadership utterances and had better CPR performance than teams that received the type of technical instruction typically provided in a traditional BLS course [12]. The improvement seen in that study was sustained at four months, even when students were placed in a new team [12]. This is particularly relevant for healthcare providers who may participate in a resuscitation but may not be part of a formal cardiac arrest team, as ad hoc teams have been shown to have lower hands-on time, delayed time to defibrillation, and make fewer leadership statements [14]. Team-based simulations may better prepare responders for team interactions during real resuscitations by providing the opportunity to practice higher-level skills.

Participants in our pilot study credited the simulation with providing a more realistic practice session than the BLS course alone, reporting that it helped them feel both more prepared and more confident. In a summary of interviews conducted with nurses after participating in real cardiac arrest resuscitations, Page & Meerabeau described how “sanitized” CPR training in a classroom can be unrealistic, with manikins not accurately conveying aspects of a real resuscitation, including sounds, skin signs, the presence of bodily fluids, and the physicality of doing compressions [15]. Although this “sanitization of CPR” may support a primary goal of lay-rescuer training, empowering would-be responders to act, this is a different training audience than healthcare providers. A simulation that is too realistic could be stressful for learners not typically exposed to the more graphic aspects of healthcare and potentially dissuade them from acting in an emergency.

However, the opposite may be true for healthcare providers with clinical experience. The realism, or fidelity, of the simulation is important to aid in the learner’s suspension of disbelief, which is their ability to look past any unrealistic aspects of the scenario and accept the simulation as reality [16]. This more realistic environment may better enable the learner to engage with the session rather than fixate on unconvincing aspects of the scenario. This phenomenon was noted by one of our study participants who reported that they “had no attachment whatsoever” to the manikin used in the BLS skills session and found the fidelity of the simulation to be more memorable. Our study participants also suggested other benefits to the realism, with one participant reporting that it helped make the idea of a real resuscitation less frightening, and another suggesting that it could potentially lessen the trauma of participating in a real resuscitation.

Beyond the benefits of added learner engagement, the high-fidelity simulation had additional practical benefits. Specifically, it helped the students understand how to apply BLS skills in a realistic setting. This is in line with previous research that demonstrated that medical students trained in crisis resource management and the use of hospital equipment more effectively performed CPR and optimized environmental factors than those who completed the traditional AHA BLS course [9]. While the aforementioned study replaced the traditional BLS course with a three-hour customized curriculum, our pilot shows the benefit of augmenting the existing Heartcode BLS course with a team-based high-fidelity simulation supplement. This format may become increasingly practical as self-directed models of BLS certification, such as the AHA’s Resuscitation Quality Improvement (RQI), increase in popularity.

It is logical that the type of simulation training described by Hunt et al. and piloted in this study would help improve the efficacy of resuscitation. While the AHA BLS course is based on deliberate practice of technical skills such as compressions, ventilation, and defibrillation, these simulations created an environment that compelled learners to practice situational skills in a similar deliberate practice methodology [9]. For example, our simulation required participants to practice lowering the bed into the CPR position, using a backboard, navigating around monitoring equipment, and managing a resuscitation in a confined space. These activities themselves required active communication between team members to coordinate these tasks, as well as the critical thinking skills to overcome obstacles that may arise in an uncontrolled setting.

This same concept could be applied with minimal modifications to the training of pre-hospital responders. For example, rather than navigating around wires in a hospital room, a pre-hospital simulation could involve initiating resuscitation measures in a simulated small bathroom or the back of an ambulance, working as a team to package an arresting patient for transport down a flight of stairs, or necessitating the use of communication skills and reassignment of roles to incorporate additional responders into the scenario as they arrive.

The student teams in our pilot were asked to lead their own debriefing after the first scenario and formulate a performance improvement plan. Within-team debriefing is not a novel concept. A previous study that randomized interdisciplinary operating room teams to an instructor-led or team-led video debriefing found no statistically significant differences between debriefing methods and the amount of improvement between groups [17]. This technique enables simulations to occur with less dedicated instructor time and may also help prepare learners to conduct post-event debriefings on their own after real resuscitation events.

Debriefing may also benefit those who did not perform the specific skill. For example, in a recent study of tobacco cessation training for medical students, students who watched other students role-play through a case and participated in a debrief reported the same self-reported benefit as those who actively participated in the cases [18]. By listening to their peers’ descriptions of challenges and takeaways, learners likely will be able to consider challenges they would not have directly experienced and add tools to their repertoire so they are prepared should they encounter them in the future.

Although this pilot study included only first-year medical students, we believe that this training approach could be useful for a wider audience. For example, many healthcare personnel who work in teams do not receive advanced life support training or work in environments where they are unable to utilize its components due to their scope of practice or because they lack the cardiac monitoring capabilities and medications emphasized in the course. These healthcare personnel would be good candidates for high-performance, team-based BLS training. Hunt et al. came to a similar conclusion, recommending the creation of an “Advanced BLS” curriculum [9]. Simulation sessions such as the one we piloted could help expand and improve the current BLS curriculum. Major training entities should consider the creation of novel BLS courses or renewal options centered around team-based high-fidelity resuscitation simulations.

Limitations and future directions

This pilot study has several limitations, including its small sample size, inclusion of only first-year medical students, single-site location, and pre-post design with no comparison group. Unfortunately, technical limitations prevented the authors from capturing objective feedback data regarding CPR quality and compression fraction, so proficiency ratings came solely from the learner’s perspective and could not be independently validated. Fortunately, previous studies have shown that cardiac arrest simulation-based training improves CPR performance [17]. Therefore, this pilot study provides a road map for future research that should include larger sample sizes and the collection of measurable CPR performance metrics such as compression fraction and time to shock to validate the learner's perspective against objective performance metrics. 

Future studies should also be conducted with different populations of learners, including medical students, nurses, physicians, advanced practice providers, pre-hospital personnel, and multidisciplinary teams. These studies should include learners with different experience levels to evaluate differences in the benefit of high-fidelity simulation-based training for novice versus experienced learners.

## Conclusions

This mixed-methods pilot study demonstrated that participation in team-based resuscitation simulations effectively augmented the AHA’s BLS course by enhancing perceived proficiency in team dynamics, including roles, responsibilities, leadership, communication, event debriefing, and the creation of performance improvement plans. For novice first-year medical students, the added realism of high-fidelity equipment and team-based scenarios helped them feel more prepared to participate in real-world resuscitations. To improve cardiac arrest outcomes, training entities should explore the use of team-based high-fidelity simulation as an adjunct to traditional BLS training and consider the development of a formal team-based BLS certification course.

## Figures and Tables

**Figure 3:**
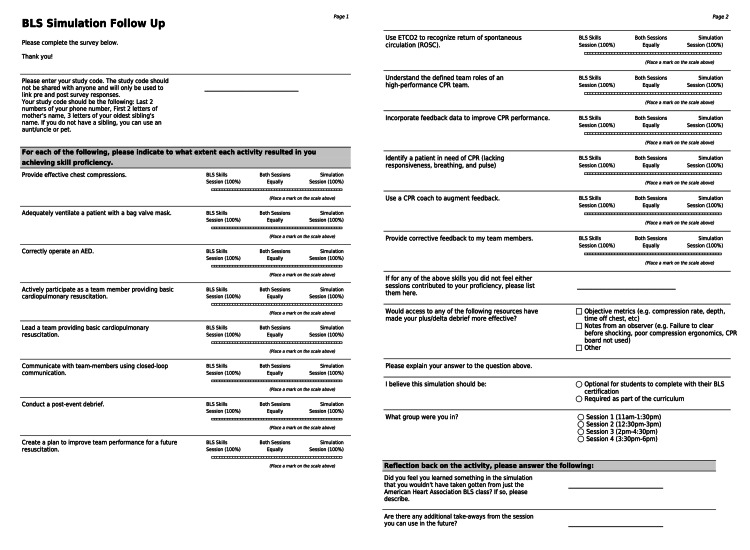
Post-session Survey
